# Impact of Surgeon Procedural Volume on Outcomes Following Cervical Disc Arthroplasty: A Retrospective Cohort Study

**DOI:** 10.7759/cureus.109560

**Published:** 2026-05-24

**Authors:** Ben Setaro, Riya Garg, Joyce Wang, Kara McConaghy, Wendy Novicoff, Xudong Li, Stephen C Ludwig, Stephen D Lockey

**Affiliations:** 1 Orthopaedic Surgery, University of Virginia School of Medicine, Charlottesville, USA; 2 Orthopaedic Surgery, University of Virginia Medical Center, Charlottesville, USA; 3 Orthopaedic Surgery, University of Virginia Health System, Charlottesville, USA; 4 Orthopaedic Surgery, University of Maryland Medical Center, Baltimore, USA

**Keywords:** cervical disc arthroplasty, healthcare utilization, postoperative complications, revision surgery, surgeon volume, surgical outcomes research

## Abstract

Purpose

The purpose of this retrospective matched cohort study was to investigate the impact of surgeon procedural volume on postoperative outcomes and healthcare utilization following CDA. Cervical disc arthroplasty (CDA) is considered a motion-preserving alternative to anterior cervical discectomy and fusion (ACDF) for addressing cervical radiculopathy and myelopathy. Increased surgeon procedural volume has been linked to improved patient outcomes and reduced costs in other orthopedic surgeries. However, limited large-scale data exist regarding the effects of surgeon experience on CDA outcomes.

Methods

Adult patients who underwent CDA between 2010 and 2022 and had database activity for at least 90 days postoperatively were identified from the PearlDiver database. Three cohorts were defined by providers’ procedural volume: high-volume, >75 procedures; mid-volume, 26 to 75 procedures; and low-volume, 11 to 25 procedures performed during the study period. Differences in baseline demographic and clinical characteristics were assessed, and logistic regression was conducted to compare postoperative complications, 30-day and 90-day emergency department (ED) visits, and readmission rates. Survival probabilities for revision and cervical reoperation were determined using Kaplan-Meier analysis.

Results

Of the 18,538 patients identified, 7,819 (42.2%) were treated by low-volume surgeons, 7,017 (37.9%) were treated by mid-volume surgeons, and 3,702 (20.0%) were treated by high-volume surgeons. There were no significant differences in 90-day complication rates or postoperative healthcare utilization in the multivariate analysis, with the exception of an increased rate of atelectasis in the high-volume cohort. Survival probabilities for revision CDA or cervical reoperation did not differ across groups (log-rank P > 0.05).

Conclusions

Surgeon procedural volume did not have a significant impact on postoperative complications, healthcare utilization, or revision rates after CDA. Nevertheless, careful attention to appropriate patient selection and surgical technique remains critical to achieving favorable outcomes.

Level of evidence

This retrospective cohort study provides Level III evidence.

## Introduction

Cervical disc arthroplasty (CDA) is a motion-preserving treatment option in the management of degenerative conditions of the cervical spine. The benefits of CDA are well documented and include faster recovery, decreased risk of adjacent segment disease, and lower reoperation rates compared with anterior cervical discectomy and fusion (ACDF) [1-3]. It is therefore not surprising that disc replacement grew by 413% between 2010 and 2020 in the United States [4]. This growth in adoption has occurred despite the fact that CDA is considered more technically demanding than ACDF [5]. For example, proper functioning of the device requires precise midline placement with meticulous contouring of the endplates to achieve “fit” and avoid subsidence. Given these technical demands, it is conceivable that surgeon experience may have an impact on outcomes after cervical disc replacement surgery.

It is well understood that surgeon experience impacts postoperative outcomes. A study investigating the relationship between hospital volume and mortality rates in the repair of aortic dissection, specifically types A and B, demonstrated lower mortality at higher-volume hospitals [6]. In addition, a systematic review of 32 reviews reported that higher procedural volume, at the surgeon or hospital level, was associated with improved outcomes such as reduced mortality, complication rates, and length of stay across a wide range of procedures, including coronary artery bypass grafting, colectomy, and cholecystectomy [7]. Moreover, another review that examined 292 studies covering a broad range of procedures found that 86.6% of the hospital volume-outcome relationships tested showed significant positive associations with reduced mortality, shorter length of stay, lower readmission rates, and fewer complications [8]. Despite these findings, there is a paucity of data on the role of surgeon experience in postoperative complications and the need for revision following cervical disc replacement.

The purpose of this investigation was to determine the impact of surgeon volume on outcomes after CDA. Specifically, the primary outcome was the rate of postoperative complications, and the secondary outcomes were the rates of healthcare utilization and revision surgery. In light of the aforementioned technical considerations when performing disc replacement, the central hypothesis of this study was that greater surgeon experience would be associated with lower rates of postoperative complications, healthcare utilization, and need for revision surgery.

## Materials and methods

Study design

This was a retrospective study utilizing the PearlDiver (PearlDiver Inc., Colorado Springs, Colorado, USA) database, which contains over 170 million patient records in the United States. As patient records were deidentified and considered Health Insurance Portability and Accountability Act (HIPAA)-compliant, this study was deemed exempt from Institutional Review Board approval.

Study population

Current Procedural Terminology (CPT) codes 22856 and 22858 were used to identify adult patients who underwent index CDA between 2010 and 2022, irrespective of the number of levels operated on [9]. Patients who did not have database activity within 90 days after their index surgery or cases performed by surgeons whose procedural volumes were 10 or fewer procedures were excluded. Using the PROVRECORDCOUNT function, three patient cohorts were created based on providers’ procedural volume during the study period: (1) high-volume, if 76 or more procedures were performed by a provider; (2) mid-volume, if 26 to 75 procedures were performed by a provider; and (3) low-volume, if 11 to 25 procedures were performed by a provider. The low-volume surgeon cohort had a minimum of 11 procedures, as the PearlDiver database was unable to index providers with fewer documented procedures.

Outcome measures

Using International Classification of Diseases, Ninth and Tenth Revision (ICD-9 and ICD-10), and CPT codes, 90-day medical complications included arrhythmia, myocardial infarction, cerebrovascular accident, atelectasis, respiratory failure, pleural effusion, pulmonary embolism, urinary retention, urinary tract infection, renal failure, and sepsis. Surgical complications within 90 days included dysphagia/dysphonia, laryngeal nerve palsy, cervicalgia, cervical kyphosis, and wound infections. Postoperative healthcare utilization outcomes included 30- and 90-day ED visits and inpatient readmissions. Revision and reoperation rates were determined at two years postoperatively, with CDA revision identified using CPT codes 22861 and 22864. Reoperation was defined in the study by Hameed Z et al. (2025) as any subsequent anterior or posterior cervical fusion, disc removal, replacement, or revision (CPT: 22551, 22554, 22552, 22600, 22864, 0098T, 0095T) [10].

Statistical analysis

Differences in patients’ baseline demographics and comorbidities were determined using Pearson’s χ² test and Welch’s t-test. Multivariate logistic regressions, controlling for age range, gender, Elixhauser Comorbidity Index (ECI), and comorbidities, were then conducted separately for the high-volume and mid-volume cohorts to calculate ORs and 95% CIs relative to the low-volume cohort. Five-year Kaplan-Meier analyses and log-rank tests were also performed to evaluate revision-free survival probability after CDA. All descriptive analyses were calculated within the R statistical software interface provided by PearlDiver. Figures were created using Python. Statistical significance was defined as p < 0.05.

## Results

**Figure 1:**
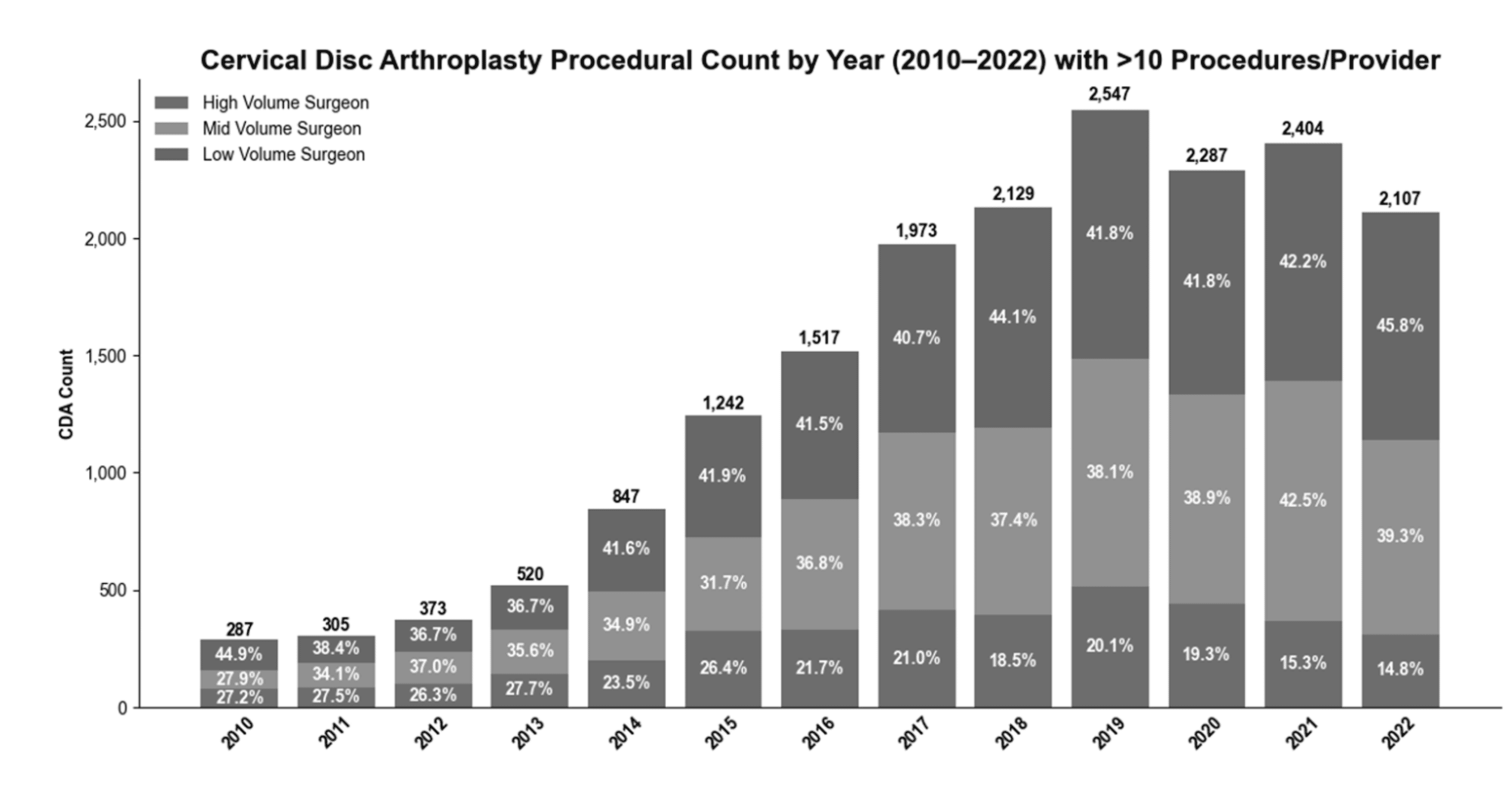
Number of CDAs performed in patients aged 18 years and older between 2010 and 2022 by providers who performed >10 procedures during the study period, stratified by procedural volume: high-volume (>75 procedures), mid-volume (26-75 procedures), and low-volume (11-25 procedures). CDA: Cervical disc arthroplasty.

A total of 18,538 CDA procedures were identified from 2010 through 2022. Of these, 7,819 (42.2%) were performed by low-volume surgeons (11-25 procedures), 7,017 (37.9%) were performed by mid-volume surgeons (26-75 procedures), and 3,702 (20.0%) were performed by high-volume surgeons (76 or more procedures). Additionally, CDA utilization steadily increased between 2010 and 2022 (Figure 1).

There were baseline differences between the three cohorts stratified by surgeon volume. On univariate analysis, the cohorts differed in terms of patient characteristics and comorbidities, geographic region, and primary insurance payer (Table 1). Patients in the high-volume surgeon cohort were slightly older on average compared with those in the mid- and low-volume groups (49.0 vs. 48.5 vs. 47.3 years, respectively; t = 43.760, P < 0.001). Patients also differed in their ECI scores, with the mid-volume cohort having a higher average comorbidity burden compared with the low- and high-volume groups (3.2 vs. 3.1 vs. 3.0, respectively; t = 12.280, P < 0.001). The study cohorts also differed significantly by geographic region (χ² = 182.710, P < 0.001) and insurance type (χ² = 85.564, P < 0.001), with low-volume surgeons more frequently operating in the southern United States and the high- and low-volume cohorts having higher rates of commercial payers. Patients in the mid-volume group tended to have greater rates of medical comorbidities, including tobacco use (22.5% vs. 21.0% vs. 18.8%, respectively; χ² = 20.083, P < 0.001), diabetes mellitus (15.9% vs. 14.4% vs. 14.8%, respectively; χ² = 6.629, P = 0.036), and hypertension (29.9% vs. 27.8% vs. 28.7%, respectively; χ² = 8.317, P = 0.016), compared with the low- and high-volume cohorts.

**Table 1:** Baseline demographic characteristics of patients who underwent CDA performed by high-volume (>75 procedures), mid-volume (26-75 procedures), or low-volume (11-25 procedures) surgeons during the study period (2010-2022). Boldface text indicates statistical significance. CDA: Cervical disc arthroplasty; ECI: Elixhauser Comorbidity Index; PVD: Peripheral vascular disease; HLD: Hyperlipidemia; HTN: Hypertension; CHF: Congestive heart failure; CAD: Coronary artery disease.

Patient characteristics	High-volume surgeons N = 3,702 (20.0%)	Mid-volume surgeons N = 7,017 (37.9%)	Low-volume surgeons N = 7,819 (42.2%)	Test statistic	P-value
Age, years, mean ± SD	49.0 ± 10.5	48.5 ± 10.2	47.3 ± 10.1	t = 43.760	<0.001
Female sex, n (%)	2,068 (55.9%)	3,904 (55.6%)	4,267 (54.6%)	χ² = 2.434	0.296
ECI, mean ± SD	3.0 ± 2.8	3.2 ± 2.8	3.1 ± 2.8	t = 12.280	<0.001
Region					
Midwest	915 (24.7%)	1,726 (24.6%)	2,165 (27.7%)	χ² = 182.710	<0.001
Northeast	578 (15.6%)	987 (14.1%)	1,055 (13.5%)		
South	1,171 (31.6%)	2,478 (35.3%)	3,068 (39.2%)		
West	1,026 (27.7%)	1,778 (25.3%)	1,479 (18.9%)		
Unknown	12 (0.3%)	51 (0.7%)	52 (0.7%)		
Insurance plan					
Commercial	3,182 (86.0%)	5,899 (84.1%)	6,785 (86.8%)	χ² = 85.564	<0.001
Government	91 (2.5%)	211 (3.0%)	195 (2.5%)		
Medicaid	117 (3.2%)	425 (6.1%)	358 (4.6%)		
Medicare	281 (7.6%)	427 (6.1%)	396 (5.1%)		
Unknown/Other	31 (0.8%)	55 (0.8%)	85 (1.1%)		
Comorbidities					
BMI 30-40	472 (12.7%)	839 (12.0%)	959 (12.3%)	χ² = 1.424	0.491
BMI ≥40	158 (4.3%)	367 (5.2%)	361 (4.6%)	χ² = 4.311	0.116
Tobacco use	695 (18.8%)	1,577 (22.5%)	1,639 (21.0%)	χ² = 20.083	<0.001
Diabetes mellitus	547 (14.8%)	1,117 (15.9%)	1,129 (14.4%)	χ² = 6.629	0.036
Alcohol use	70 (1.9%)	165 (2.4%)	185 (2.4%)	χ² = 2.937	0.23
Chronic pulmonary disease	40 (1.1%)	83 (1.2%)	111 (1.4%)	χ² = 2.890	0.236
PVD	103 (2.8%)	169 (2.4%)	187 (2.4%)	χ² = 1.801	0.406
HLD	746 (20.2%)	1,439 (20.5%)	1,562 (20.0%)	χ² = 0.656	0.72
HTN	1,063 (28.7%)	2,098 (29.9%)	2,170 (27.8%)	χ² = 8.317	0.016
CHF	34 (0.9%)	73 (1.0%)	88 (1.1%)	χ² = 1.049	0.592
CAD	209 (5.6%)	358 (5.1%)	387 (4.9%)	χ² = 2.540	0.281

**Figure 2:**
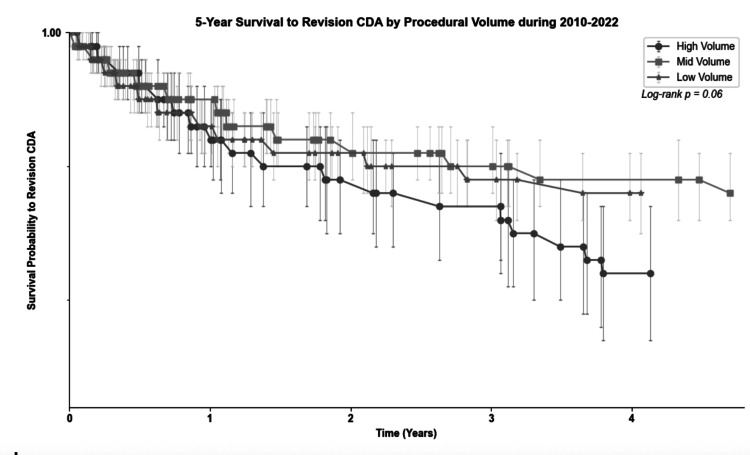
Five-year Kaplan-Meier survival curves for patients undergoing CDA performed by high-, mid-, and low-volume surgeons, with revision CDA as the endpoint. CDA: Cervical disc arthroplasty.

**Figure 3:**
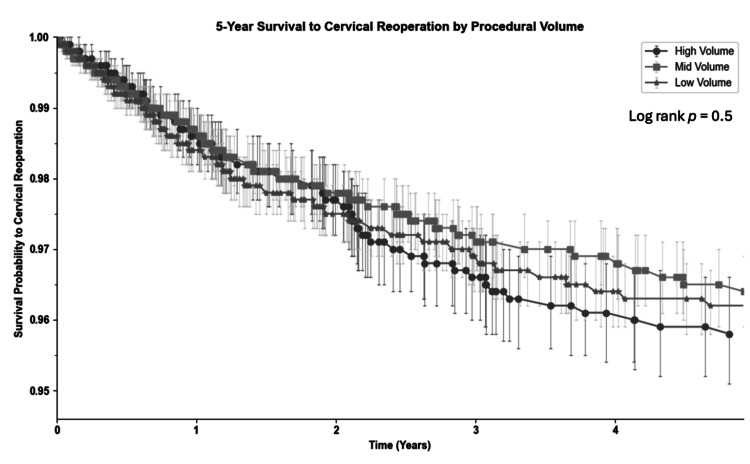
Five-year Kaplan-Meier cervical reoperation-free survival curves for patients undergoing CDA performed by high-, mid-, and low-volume surgeons. CDA: Cervical disc arthroplasty.

In multivariate regression, the 90-day postoperative outcomes revealed no differences in rates of medical complications between groups, with the exception of atelectasis (Table 2). The high-volume surgeon cohort had greater rates of atelectasis compared with the mid- and low-volume cohorts (0.4% vs. 0.3% and 0.3%, respectively; z = 16.438, P < 0.001). Further, there were no statistically significant differences in 90-day surgical complication rates or postoperative healthcare utilization based on surgeon experience. Common surgical complications, such as cervicalgia and cervical radiculopathy, occurred in 15-26% of patients but did not differ significantly by surgeon procedural volume. Notably, there was no significant difference in rates of revision CDA and cervical reoperation at two years postoperatively. Kaplan-Meier analysis showed no significant differences in survival to revision CDA or cervical reoperation by surgeon procedural volume (Figures 2-3; log-rank P > 0.05).

**Table 2:** Univariate analysis of postoperative complications and healthcare utilization among patients who underwent CDA, stratified by procedural volume. Boldface text indicates statistical significance. CDA: Cervical disc arthroplasty; CVA: Cerebrovascular accident; PE: Pulmonary embolism.

90-day postoperative complications	High-volume surgeons N = 3,702		Mid-volume surgeons N = 7,017		Low-volume surgeons N = 7,819		Test statistic	Univariate p-value
	N	%	N	%	N	%		
Medical complications								
Arrhythmia	83	2.20%	150	2.10%	183	2.30%	z = 0.693	0.707
Myocardial infarction	4	0.10%	8	0.10%	12	0.20%	z = 0.610	0.737
CVA	3	0.10%	12	0.20%	14	0.20%	z = 1.699	0.428
Atelectasis	14	0.40%	21	0.30%	24	0.30%	z = 16.438	<0.001
Respiratory failure	5	0.10%	14	0.20%	24	0.30%	z = 3.721	0.156
Pleural effusion	6	0.20%	4	0.10%	10	0.10%	z = 2.985	0.225
PE	0	0.00%	3	0.00%	6	0.10%	z = 3.127	0.209
Urinary retention	17	0.50%	22	0.30%	44	0.60%	z = 5.167	0.076
UTI	57	1.50%	110	1.60%	116	1.50%	z = 0.179	0.914
Renal failure	10	0.30%	25	0.40%	29	0.40%	z = 0.782	0.677
Sepsis	4	0.10%	3	0.00%	6	0.10%	z = 1.559	0.459
Surgical complications								
Cervical radiculopathy	560	15.10%	1,140	16.20%	1,305	16.70%	z = 4.531	0.104
Dysphagia or dysphonia	96	2.60%	132	1.90%	173	2.20%	z = 5.962	0.051
Laryngeal nerve palsy	8	0.20%	18	0.30%	21	0.30%	z = 0.278	0.87
Cervicalgia	952	25.70%	1,783	25.40%	2,004	25.60%	z = 0.150	0.928
Cervical kyphosis	3	0.10%	20	0.30%	19	0.20%	z = 4.623	0.099
Wound infection	6	0.20%	10	0.10%	19	0.20%	z = 2.157	0.34
Revision CDA, 2 years	40	1.10%	54	0.80%	68	0.90%	X 2 = 2.708	0.258
Cervical reoperation, 2 years	80	2.20%	138	2.00%	176	2.30%	X 2 = 5.337	0.481
Postoperative healthcare utilization								
ED visit, 30 days	169	4.60%	292	4.20%	322	4.10%	z = 1.349	0.509
ED visit, 90 days	302	8.20%	507	7.20%	554	7.10%	z = 4.510	0.105
Inpatient readmission, 30 days	24	0.60%	61	0.90%	69	0.90%	z = 1.876	0.931
Inpatient readmission, 90 days	57	1.50%	103	1.50%	112	1.40%	z = 0.200	0.905

## Discussion

As of 2025, several ten-year outcome studies have demonstrated favorable results from a variety of disc replacement systems [11-13]. While the technical considerations vary slightly between devices, careful patient selection, proper implant placement, and meticulous bony preparation are necessary to achieve the intended outcome. Contrary to the study hypothesis, the results of this analysis suggest that surgeon volume did not have a significant impact on outcomes following CDA. These findings stand in contrast to other reports in the literature on the impact of surgeon experience on postoperative outcomes.

Surgeon volume is associated with lower complication rates in orthopaedic surgery. Prior studies have demonstrated superior outcomes in patients treated by high-volume surgeons following joint reconstruction, such as total hip, knee, and shoulder arthroplasty [14-17]. Specifically, Malik AT et al. found that increasing surgeon volume was associated with a shorter length of stay, lower costs, and lower dislocation rates following total hip replacement [15]. Additionally, Harkin W et al. found a similar relationship in total shoulder arthroplasty, reporting that high-volume surgeons had significantly lower overall postoperative complication rates [16].

Surgeon experience has also been shown to consistently improve outcomes in spinal surgery [18-22]. For example, a review by Farjoodi P et al. found that high-volume surgeons were associated with lower mortality and complication rates following lumbar spine surgery [18]. Similarly, a meta-analysis by Malik AT et al. demonstrated that greater surgeon case volume was associated with lower morbidity and mortality, shorter hospital stays, fewer readmissions, and lower hospital costs in patients undergoing spinal surgeries such as ACDF and circumferential cervical fusion [19]. Additionally, Patel MR et al. found that, for ACDF, high procedural volume was associated with decreased surgical time, blood loss, and length of postoperative stay, in addition to improved clinical outcomes in physical function, disability, and pain [20].

One potential explanation for the results of this study is that the minimum of 11 disc replacements performed may be near the end of the learning curve for the procedure. However, a study by Federico VP et al. estimated the learning curve for cervical disc replacement to be approximately 14 cases [23]. During the learning phase, the authors reported that patients experienced longer operative times, increased postoperative narcotic consumption, and worse postoperative Visual Analog Scale (VAS) scores. In the present study, low-volume surgeons were categorized as those having 11 to 25 prior cases of experience, which may fail to capture all surgeons within the learning curve for disc replacement. The lack of differences in outcomes between cohorts may also reflect the relatively standardized indications, operative technique, and perioperative protocols associated with cervical disc arthroplasty [24].

The study results indicate that the high-volume surgeon cohort was associated with greater rates of atelectasis. These findings may be explained by increased detection bias in the high-volume surgeon cohort. High-volume surgeons may employ more refined perioperative protocols, including more sensitive postoperative imaging or documentation practices, which could result in higher reported rates of atelectasis within this group. This explanation is consistent with the lack of significant differences in rates of other pulmonary complications, including respiratory failure and pleural effusion, across the study cohorts. Furthermore, the increased occurrence of atelectasis in the high-volume cohort may not be clinically meaningful due to the overall low rates observed in this study.

There are several important limitations to this analysis. As with all claims database studies, miscoding or variation in coding practices could affect data quality. According to the Medicare Fee-for-Service Improper Payment Report, coding errors were present in 0.9% of claims in 2023 [25]. Moreover, PearlDiver provided limited granularity because of the lack of key clinical details, including radiographs, operative reports, disability scores, and other patient-reported outcome measures. Also of note, the study cohort was not evenly distributed across surgeon volume categories, with 42.2% of procedures performed by low-volume surgeons. Additionally, the study’s inclusion criteria did not exclude patients undergoing multilevel CDA, who may be more clinically complex to manage and predisposed to developing more complications. Lastly, the minimum number of procedures available within the PearlDiver database was 11, which may have failed to capture surgeons within the learning curve.

Despite these limitations, this study offers novel insights into the safety of CDA and the consistency of outcomes across surgeon experience levels. These results suggest that CDA may have a relatively flat learning curve, with comparable results following 10 index procedures [26]. Consequently, surgeons with less experience may consider offering cervical disc replacement in appropriately selected cases. The findings are also consistent with the previously suggested number of 14 disc replacement operations within the learning curve. As a result, this study may inform training programs on case volume targets at their institutions for graduates to achieve technical proficiency.

## Conclusions

In summary, this retrospective study found no statistically significant effect of surgeon procedural volume on complications, healthcare utilization, or survival to subsequent revision or reoperation after cervical disc arthroplasty. Less experienced surgeons may still consider offering disc replacement as an option for appropriate candidates. However, these results should be interpreted cautiously and replicated in future prospective analyses.
